# Phosphorus Poisoning

**Published:** 1899-03-25

**Authors:** 


### The Hospital

A Journal of

The Medical Sciences and Hospital Administration.

Vol. XXV.—No. 652.] Edited by Sir Henry Burdett, K.C.B., and Solomon C. Smith, M.D., M.R.C.P. [March 25, 1899.

### PHOSPHORUS POISONING.

The official investigation by Professors Thorpe and Oliver and Dr. Cunningham into the subject of the accidental poisoning by phosphorus of persons employed in the manufacture of matches has been brought to a termination, and their report, with an important introductory memorandum by Dr. Arthur Whitelegge, the Chief Inspector of Factories, was published as a Blue Book on Thursday last. The whole question of dangerous occupations is one of great importance, and is, unfortunately, one that may readily be made to lend itself to the purposes of agitators. Speaking generally, the danger in such cases is largely preventable, and is due in only slight degree to conditions which are essential to the prosecution of the industry involved. As a rule it will be found that something may be done by the improvement of buildings, of processes, and of machinery, and that a good deal more may be done by the observance of precautions for which the employers must, indeed, provide the means, but which only the workers themselves can carry into effect. It is at this point, again speaking generally, that the chief difficulties have arisen. There have, of course, been examples of badly constructed and badly equipped factories, but there have been many more of personal neglect by the hands employed, who only too frequently have appeared to lack the power of that "scientific exercise of the imagination" which seems to be one of the most essential conditions for enabling human beings to realise the bearing of a given course of conduct upon the probable future of their lives. A certain amount of intellectual development, as distinguished from mere schooling, is required in order to enable a worker in lead, or a worker in phosphorus, to understand practically that the consequences of eating with unwashed hands, although possibly remote, are none the less certain; and the stupidity or carelessness of the adult in such matters is only too apt to resemble that which would not be abnormal in a child. A second difficulty on the side of the workpeople arises from the fact that the exercise of dangerous trades appears in itself to be a source of recklessness, not only as a result of familiarity, but also because high wages are paid, and it is feared that a diminution of danger might bring about a reduction of the scale. Notwithstanding these well-known facts, agitators who trade upon grievances, and who endeavour to convert them into some form of political capital, are prone to fasten now and again upon the alleged evils attendant upon some particular industry, and to derive from them the raw materials for the manufacture of an indictment against some class or party to which they are opposed. It is always desirable to possess the means of estimating such attempts at their true value, and hence such reports as that which forms the basis of our present observations cannot fail to be eminently useful to the community.

The dangers incidental to match making are entirely due to the use of the "yellow" or ordinary form of phosphorus, no substitute for which, in the manufacture of "strike anywhere" matches, has yet been discovered, while the demand for these matches in the United Kingdom is so considerable that to prohibit them, as has been done in Denmark, would be to destroy an important native industry, and, unless importation were also forbidden, to throw it into the hands of foreigners. For matches which strike only on the box, or rather for the composition on which they strike, the "red," or allotropic form of phosphorus is employed, and this is perfectly harmless. The dangers of the yellow form are due partly to its introduction into the mouth by eating with unwashed hands, and partly to the inhalation of its fumes; and these dangers are declared to be practically non-existent if the teeth be sound, and if proper care be taken of them. Even in the more wealthy classes, however, the prescribed conditions are very seldom fulfilled; and some very remarkable statements have recently been made as to the results of a systematic examination of the teeth of the boys at a great public school. It would seem to be imperatively necessary, if safety in match-making is to be attained, that the manufacturers should insist upon an effective examination of the teeth of all candidates for employment, and at regular intervals of those of all persons employed, with a view to the temporary or permanent exclusion of any whose teeth were carious, or in whom the wounds left by extractions had not had time to become completely and soundly healed. Dr. Whitelegge observes that "compulsory dentistry, even if provided at the expense of the employer, is not likely to be welcomed by all the persons employed," but he nevertheless insists upon its necessity, and the report gives good ground for believing that, if this requirement be fulfilled, if ample facilities for cleanliness be not only given but utilised, and if modern improvements be adopted for the diminution of fuming and for the performance of the more dangerous processes by machinery, the manufacture of matches, even with yellow phosphorus, may be converted into a reasonably safe form of industry, or, at all events, into one which will afford no ground for injurious imputations upon those who provide the capital by means of which it is carried into effect.

